# Roles of the *APETALA3–3* ortholog in the petal identity specification and morphological differentiation in *Delphinium anthriscifolium* flowers

**DOI:** 10.1093/hr/uhae097

**Published:** 2024-04-09

**Authors:** Peng Zhang, Yanru Xie, Wenjie Xie, Li Li, Hanghang Zhang, Xiaoshan Duan, Rui Zhang, Liping Guo

**Affiliations:** College of Horticulture, Northwest A&F University, Yangling 712100 Shaanxi, China; College of Horticulture, Northwest A&F University, Yangling 712100 Shaanxi, China; College of Horticulture, Northwest A&F University, Yangling 712100 Shaanxi, China; College of Horticulture, Northwest A&F University, Yangling 712100 Shaanxi, China; College of Horticulture, Northwest A&F University, Yangling 712100 Shaanxi, China; College of Forestry, Northwest A&F University, Yangling 712100 Shaanxi, China; College of Horticulture, Northwest A&F University, Yangling 712100 Shaanxi, China; College of Horticulture, Northwest A&F University, Yangling 712100 Shaanxi, China

## Abstract

The genus *Delphinium* (Ranunculaceae) with its unique and highly complex floral structure is an ideal system to address some key questions in terms of morphological and evolutionary studies in flowers. In *D. anthriscifolium*, for example, the original eight petal primordia differentiate into three types at maturity (i.e., two dorsal spurred, two lateral flat, and four ventral reduced petals). The mechanisms underlying their identity determination and morphological differentiation remain unclear. Here, through a comprehensive approach combining digital gene expression (DGE) profiles, *in situ* hybridization, and virus-induced gene silencing (VIGS), we explore the role of the *APETALLATA3–3* (*AP3–3*) ortholog in *D. anthriscifolium*. Our findings reveal that the *DeanAP3–3* not only functions as a traditionally known petal identity gene but also plays a critical role in petal morphological differentiation. The *DeanAP3–3* gene is expressed in all the petal primordia before their morphological differentiation at earlier stages, but shows a gradient expression level difference along the dorsventral floral axis, with higher expression level in the dorsal spurred petals, intermediate level in the lateral flat petals and lower level in the ventral reduced petals. VIGS experiments revealed that flowers with strong phenotypic changes showed a complete transformation of all the three types of petals into non-spurred sepals. However, in the flowers with moderate phenotypic changes, the transformation of spurred petals into flat petals is associated with moderate silencing of the *DeanAP3–3* gene, suggesting a significant impact of expression level on petal morphological differentiation. This research also shed some insights into the role of changes in gene expression levels on morphological differentiation in plants.

## Introduction

Flowers, unique to angiosperms, exhibit a wide diversity in size, morphology, structure, and color, with a particularly rich abundance in the basic structural variation. The basic structure of a flower, represented at the types, number, arrangement, fusion, characteristics, and symmetry of its floral components (e.g., sepals, petals, stamens, and carpels), plays an integral role in its overall form [1]. Any change in one or more of these aspects can have a profound effect on the flower’s structure [1]. This, in turn, allows flowers to be categorized as simple or complex based on their basic structure. Simple flowers have a small and fixed number of simple floral organs or a larger number while maintaining radial symmetry, such as *Arabidopsis* (Brassicaceae), *Papaver* (Papaveraceae), and the Chloranthaceae [2–4]. Conversely, complex flowers, manifest bilateral or asymmetric symmetry or possess specialized floral organs, as typified by taxa such as the Fabaceae, Lamiaceae, Ranunculaceae, Orchidaceae, and Zingiberaceae [3–5]. Complex flowers, widespread among angiosperms, hold substantial adaptive significance and make these plants essential resources for breeding ornamental plants [3, 5]. Therefore, an in-depth exploration into the molecular mechanisms that underpin establishment and diversification of floral basic structures is imperative.

**Figure 1:**
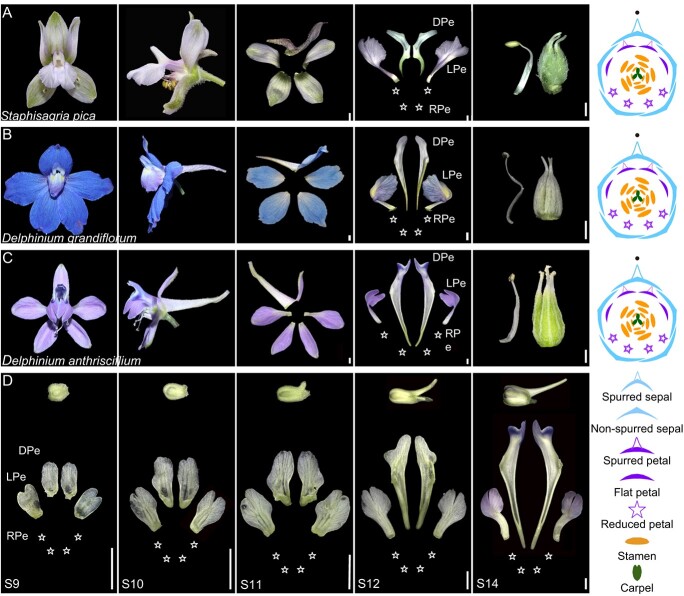
Floral structures of the Delphinieae and the petal differentiation in *Delphinium anthriscifolium*. (**A**–**C**) Front views (first column) and side views (second column) of mature representative Delphinieae species flowers, along with disassembled floral organs: sepals (third column), petals (fourth column), and stamens with carpels (fifth column). Perianth diagrams in the last column illustrate flower structure. (**D**) Petal differentiation in *Delphinium anthriscifolium* floral buds from stages S9 to S14, showing side views of floral buds (top), and front views of dorsal spurred petals (DPe), lateral petals (LPe), and reduced petals (RPe, indicated by white five-pointed stars). Scale bars: 1.0 mm.

One of the most foundational research achievements in the establishment of flower basic structure is the proposal of the ABC model of flower development: A function genes specifies sepals; A + B function genes, petals; B + C function genes, stamens; and C function genes, carpels [6–8]. The ABC model is generally conserved, with most floral organ identity genes responsible for A, B, and C functions predominantly belonging to the MADS-box gene family, such as *APETALA1* (*AP1*) and *AGAMOUS-LIKE6* (*AGL6*), *APETALA3* (*AP3*) and *PISTILLATA* (*PI*), and *AGAMOUS* (*AG*) subfamily members [9–11]. The ABC model also provides a theoretical foundation and research clues for understanding diversification of flower basic structure. In numerous taxa, alterations in floral organ types and compositions are closely associated with the modification of floral organ identity genes. For instance, the specialized orchid labellum (Orchidaceae) results from the duplication and neofunctionalization of a B-function gene, *AP3*, coupled with a reduction in its expression domain [12]. Similarly, the formation of petal-like tepals in lilies (Liliaceae) plants, such as *Lilium brownii* and tulips (*Tulipa gesneriana*), is caused by heterotopic expression of B-functional genes in the outer two whorls of floral organs [13, 14]. In the Ranunculaceae, the independent loss of petals is often associated with the ‘inactivation’ of B functional genes, resulting in the transformation of petals into sepals, or the expansion of C functional genes into the petal whorl (leading to the transformation of petals into stamens) [15, 16]. While these studies have provided a crucial foundation for comprehending the molecular mechanisms behind establishment and diversification of flowers with relatively simple structures, they fall short of explaining how complex flowers form and evolve.

The Delphinieae tribe (Ranunculaceae) are an ideal group for addressing these questions, given the extraordinary complexity of their floral structures. Characterized by a spiral arrangement of floral organs and a nearly bilateral symmetry, the flowers in this tribe are often referred as almost monosymmetric spiral flowers exemplifying a form of ‘irregular’ flowers [17] (Fig. 1A–C). In particular, a significant factor contributing to the heightened complexity of the flower basic structure in this tribe is the presence of different petal types and their combinations [4, 18, 19]. For instance, in the *Delphinium* genus, most plants have flowers with two dorsal spurred petals (SP), two lateral flat petals (FP), and four ventral reduced petals (RP), forming a ‘2 + 2 + 4’ pattern (Fig. 1B and C); however, *Delphinium consolida* flowers consist of two fused dorsal spurred petals and six reduced petals, forming a ‘(1 + 1) + 6’ pattern; the *Aconitum* and *Gymnaconitum* genera showcase a ‘2 + 6’ petal arrangement with two dorsal spurred petals and six reduced petals. The basal genus *Staphisagria* also follows a ‘2 + 2 + 4’ petal combination, akin to *Delphinium* [18, 20] (Fig. 1A). Therefore, investigating the mechanisms governing the petal morphological differentiation in this tribe is crucial for comprehending the diversity of fundamental plant structures and the formation of complex flowers.

Previous studies on the Ranunculaceae have significantly advanced our understanding of the development of complex floral structures. Studies have demonstrated that the *AP3* class genes in this family underwent two successive gene duplications, resulting in the emergence of three distinct copies, namely *AP3–1*, *AP3–2*, and *AP3–3* [16, 20–22]*.* It has been established that the *AP3–3*-lineage genes specifically determine the petal identity due to subfunctionalization, exhibiting highly specific expression in petals of the Ranunculaceous flowers. Mutations or silencing of this gene can lead to the transformation of petals into sepals [4, 11, 21–24]. A recent study in a *Delphinium* species, *Delphinium ajacis*, indicates that the *DeajAP3–3* gene functions specifically as a spurred petal identity gene, while has no roles in reduced petals, which was associated with its differential expression in levels along the dorsiventral axis of flower [4]. However, the ‘(1 + 1) + 6’ combination of *D. ajacis* petals is still relatively simpler compared with other lineages of the Delphinieae tribe. In *Delphinium anthriscifolium* flowers, for example, their petals display a ‘2 + 2 + 4’ combination (Fig. 1C), representing a more complex floral structure, but the mechanisms underlying their morphological differentiation remain unclear.

To gain a deeper understanding of the molecular mechanisms underlying morphological differentiation of petals in complex flowers of the Delphinieae, we are using the representative plant *D. anthriscifolium* as our research material. Through morphological, comparative transcriptome analysis, and molecular biology approaches, we aim to reveal the distinct morphological characteristics and developmental trajectories of three petal types, identify key genes and molecular processes controlling the morphological differentiation of different petal types, and validate their functional characteristics. These research findings are expected to lay the foundation for understanding the molecular mechanisms of complex flower formation and diversification in the Delphinieae and, more broadly, in angiosperms. Additionally, they will provide crucial theoretical insights for ornamental horticulture breeding.

## Results

### Morphological differentiation of petals

To understand the morphological and developmental bases of petal differentiation, we chose *D. anthriscifolium* as a representative for morphological and anatomical studies. According to Zhang *et al.* (2022), the development of *D. anthriscifolium* flowers, involves 16 stages, from the initiation of floral meristems to maturity [25]. Prior to stage 9 (S9), all petal primordia initiate as spherical shape, then rapidly elongate and expand into shapes resembling flattened rectangles, exhibiting no obvious morphological differentiation. At S9, the pair of dorsal petals begin to show basal depressions, while that of lateral petals develop bifurcated lobes, and the development of ventral petals ceases, indicating the onset of dorsal-ventral differentiation of the flowers (Fig. 1D). By S10, the depressions of the dorsal petals grows into spurs, and the lateral petals continue to enlarge, while the ventral petal primordia cease to grow (Fig. 1D). At S11, both the dorsal spurred petals and lateral flattened petals undergo rapid development, with progressive spur elongation specifically observed in the dorsal spurred petals (Fig. 1D). In S12, the spurs of the dorsal petals have doubled in length compared to S11, and the lateral petals start asymmetric folding (Fig. 1D). By S14, the petals form their final structure, and pigmentation gradually begins (Fig. 1D).

### Floral organ identity genes associated with petal morphological differentiation

**Figure 2:**
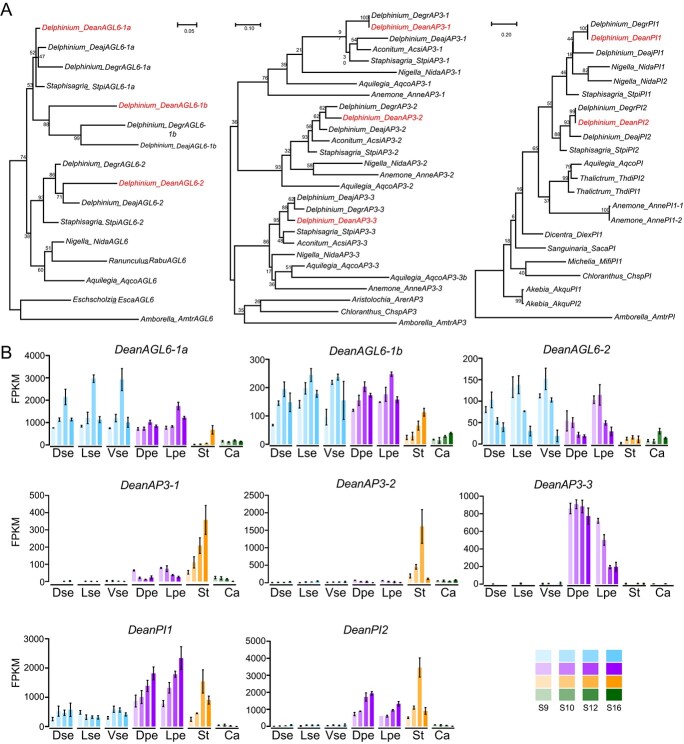
Phylogenetic analysis and spatiotemporal expression patterns of putative A and B function genes in *Delphinium anthriscifolium*. (**A**) Phylogenetic trees for *AGL6, AP3*, and *PI* gene lineages. Genes from *Delphinium anthriscifolium* are highlighted. Branch lengths represent the number of nucleotide substitutions per site. (**B**) Spatiotemporal expression patterns of *AGL6, AP3*, and *PI* lineage genes as determined by DGE analysis. The bottom right inset depicts sepals, petals, stamens, and carpels across developmental stages S9–S16, with color intensity indicating the stage. Ca, Carpel; DPe, dorsal spurred petal; Dse, dorsal spurred sepal; LPe, lateral petal; Lse, lateral sepal; St, Stamen; Vse, ventral sepal.

To understand the molecular mechanisms underlying the morphological differentiation of petals in *D. anthriscifolium* flowers, we cloned putative A- and B-function genes implicated in specifying the petal identity. As a result, three, three, and two *AGL6*, *AP3*, *PI* lineage genes were obtained, respectively. Phylogenetic analysis revealed that the three lineage genes all underwent duplication events (Fig. 2A). Specifically, two successive Delphinieae-specific duplication events led to the generation of the *DeanAGL6-1a*, *DeanAGL6-1b*, and *DeanAGL6–2*. For *DeanAP3* lineage, *DeanAP3–3*, together with its two closest paralogs, *DeanAP3–1* and *DeanAP3–2*, were generated through two gene duplication events predating the divergence of the Ranunculaceae. In the case of *DeanPI*, independent duplication events occurred subsequent to the last common ancestor of all analysed species, generating *DeanPI1* and *DeanPI2* genes.

We next performed digital gene expression (DGE) analysis for the three lineage genes (Fig. 2B; Fig. S1, see online supplementary material). The results revealed that all three *DeanAGL6*-like genes exhibited higher expression levels in both sepals and petals relative to carpels and stamens across four developmental stages (S9, S10, S12, and S16) of flowers. Notably, the *DeanAGL6-1a* gene exhibited significantly higher expression levels compared to the other two paralogs. For the *DeanPI*-like genes, the *DeanPI1* was mainly expressed in sepals, petals, and stamens, while the *DeanPI2* was exclusively expressed in petals and stamens. Moreover, the two genes demonstrated increased expression levels in petals through development. For the *DeanAP3*-like genes, the expression patterns of the three genes varied distinctly. *DeanAP3–1* exhibited higher expression levels in both petals and stamens, particularly in stamens, while *DeanAP3–2* showed its highest expression in stamens. *DeanAP3–3*, a key petal identity gene, was predominantly expressed in petals as expected [16, 21, 26]. However, this gene drew our special attention due to its higher expression in spurred petals than in lateral petals during stages S9–S16, coinciding with distinct morphological changes (Fig. 2B; Fig. S1, see online supplementary material). Therefore, we consider that the expression level differences of *DeanAP3*–3 may be instrumental in the morphological differentiation of petals in *D. anthriscifolium*.

### Effects of *DeanAP3–3* on the morphological differentiation of petals

**Figure 3:**
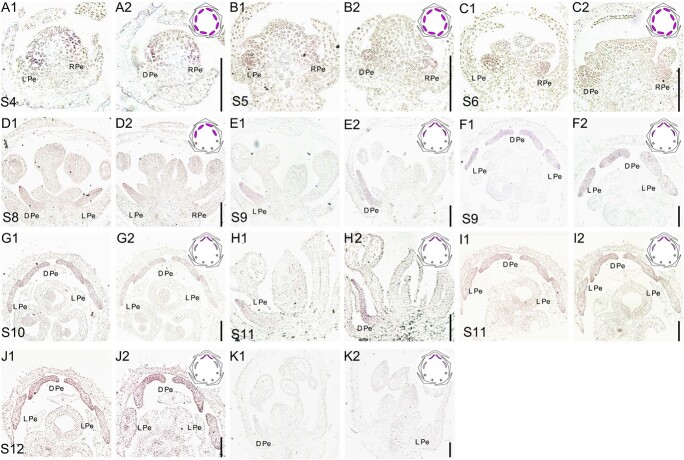
Spatiotemporal expression of *DeanAP3–3 in Delphinium anthriscifolium* flowers. Serial sections of the same floral bud or petal are labeled as serial numbers. The images F, G, I, and J shows the transverse section of the floral bud, while the remaining images display longitudinal sections. Expression models of *DeanAP3–3* gene are showed in the upper right corner of each set of pictures, with the color illustrating the expression domain. The final images (K) show the results using sense probes. DPe, dorsal spurred petal; LPe, lateral petal; RPe, reduced petal. Scale bars: 200 μm.

To verify the above hypothesis on *DeanAP3–3* gene’s role in petal differentiation in *D. anthriscifolium,* we firstly conducted *in situ* hybridization experiments to gain precise insights into its spatiotemporal expression. At S4 of floral development, its expression was specifically detected in all petal primordia, with no obvious difference among different types of petals (Fig. 3A). This uniform expression pattern persisted until S6, prior to the eight petal primordia differentiated (Fig. 3B–C). By S8, with the differentiation of petal primordia along the dorsoventral axis, its expression remained high in the two dorsal and two lateral petals, whereas the ventral reduced petals showed only faint expression (Fig. 3D). At S9, coinciding with the onset of petal morphological differentiation, *DeanAP3–3* still maintained high expression levels in the dorsal and lateral petals, but no expression signal was detected in the degenerated petals (Fig. 3E–F). During S10 and S11, the *DeanAP3–3* gene continued to exhibit strong expression signals in the dorsal spurred petals but showed weakened expression signals in lateral flat petals (Fig. 3G–I). By S12, the expression signal of the *DeanAP3–3* gene in dorsal spurred petals was stronger than that in lateral flat petals (Fig. 3J), aligning with the morphological differentiation of petals.

**Figure 4:**
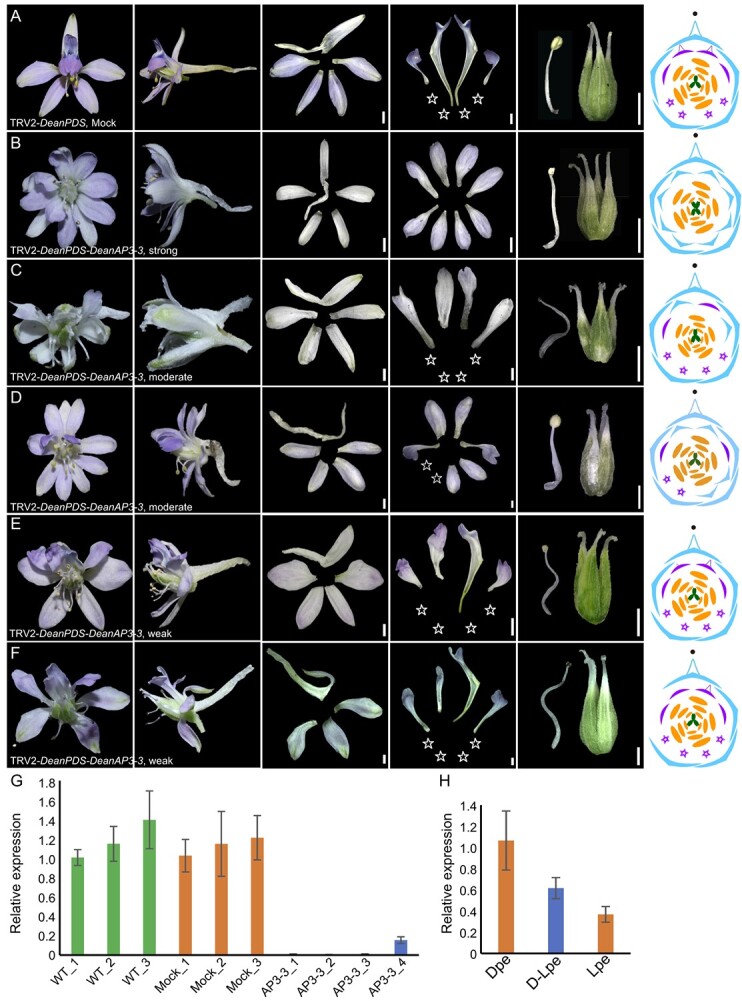
Function of *DeanAP3–3* as revealed by virus-induced gene silencing. (**A**–**F**) Flowers in the mock (**A**), TRV2-*DeanPDS*-*DeanAP3–3* treated plants with strong (**B**), moderate (**C** and **D**), and weak (**E** and **F**) phenotypic changes. Each panel, from left to right, presents the front and side views of the whole flower, followed by dissections revealing the anatomical structure of sepals, petals (with reduced petals marked by a white five-pointed star), and stamens and carpels. The floral diagram in the last column illustrates the altered structure of flowers. (**G**) Relative expression levels of *DeanAP3–3* in S12 floral buds of WT, mock and TRV2-*DeanPDS*-*DeanAP3–3* treated plants. AP3–3_1–3 are flowers with strong phenotypic changes, and AP3–3_4 are flowers with moderate phenotypic changes. (**H**) Relative expression levels of *DeanAP3–3* in dorsal (DPe) and lateral petals (LPe) of mock plants, and in lateral petals (D-LPe, dorsal petals transformed) of TRV2-*DeanPDS*-*DeanAP3–3* treated plants. Scale bars: 1000 μm.

To further investigate the role of *DeanAP3–3* in *D. anthriscifolium*, we utilized the virus-induced gene silencing (VIGS) technique with the Tobacco rattle virus (TRV) construct, TRV2-*DeanPDS*-*DeanAP3–3*, with *DeanPDS* as a silencing efficacy marker (Fig. 4; Figs S2 and S4, see online supplementary material). In contrast to the TRV2-*DeanPDS* treated plants (mock; Fig. 4A), plants subjected to TRV2-*DeanPDS*-*DeanAP3–3* exhibited various phenotypic alterations. Plants with strong phenotypic changes showed a complete transformation of all the three types of petals—i.e., spurred, flat, and reduced petals—into non-spurred sepals, with other floral organs minimally affected (Fig. 4B; Fig. S3, see online supplementary material). Interestingly, flowers with moderate phenotypic changes could be categorized into two groups. In the first group, the dorsal spurred petals were completely transformed into flat petals (Fig. 4C; Fig. S3, see online supplementary material). In the second group, the dorsal spurred petals manifested as intermediates between sepals and flat petals, and some ventral reduced petals converted into sepals (Fig. 4D; Fig. S3, see online supplementary material). In plants with weak phenotypic changes, only dorsal spurred petals showed a transition towards flat petal characteristics, while the lateral flat petals are minimally affected (Fig. 4E and F; Fig. S3, see online supplementary material).

Corresponding to these morphological shifts, significantly reduced *AP3–3* expression levels were observed in petals with strong phenotypic changes compared to WT and mock (Fig. 4G), indicative of *DeanAP3–3*’s pivotal role in identity determination of all the petals*.* However, it is interestingly to find that in the group of the weak phenotypic changes, the expression of *DeanAP3–3* in dorsal petals that transformed from the spurred to flat petals showed an intermediate level between those in the true dorsal spurred and lateral flat petals (Fig. 4H).

**Figure 5:**
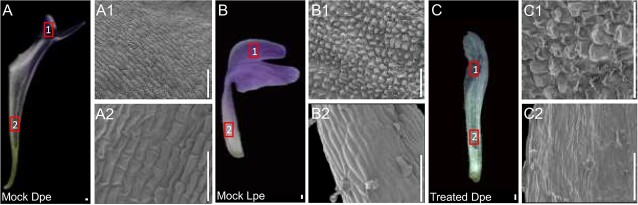
Morphological characteristics in *Delphinium anthriscifolium* petals. (**A**) Dorsal petal of a mock-treated plant with close-ups of the epidermal cells in the distal (A1) and proximal (A2) regions, as indicated by square regions 1 and 2, respectively. (**B**) Lateral petal of a mock-treated plant with close-ups of the epidermal cells in the distal (B1) and proximal (B2) regions, as indicated by square regions 1 and 2, respectively. (**C**) Dorsal petal of a plant exhibiting weak phenotypic changes, with detailed views of the distal (C1) and proximal (C2) epidermal cells, as indicated by square regions 1 and 2, respectively. Scale bars: 100 μm

To verify that dorsal spurred petals in plants with weak phenotypic changes indeed transitioned towards flat petal characteristics, we performed SEM analysis and compared the epidermal cell types on the ventral side of the dorsal spurred petal and flat petal in mock plants, as well as the dorsal petal in plants exhibiting weak phenotypic changes. In the proximal region (domain 2), all three petal types were characterized by elongated epidermal cells (Fig. 5A–C). In the distal region (domain 1), the epidermal cells of spurred petals in mock plants were elongated, whereas the flat petals displayed conical cells (Fig. 5A and B). Interestingly, the dorsal petals of plants with weak phenotypic changes also had conical epidermal cells in the distal region, aligning with the cell type observed in flat petals of mock plants (Fig. 5A–C). This suggests that in plants with weak silencing of the *AP3–3* gene, the dorsal spurred petals undergo a transformation into flat petals.

## Discussion

In angiosperms, *AP3*-like genes are pivotal in the determination of petal and stamen identities. Within the Ranunculaceae family, the *AP3* genes have expanded via two duplication events, yielding three distinct copies, with *AP3–3* being crucial for specifying petal identity [4, 11, 16, 20–24, 26, 27]. Unlike the broader expression of *AP3–1* and *AP3–2*, *AP3–3* is uniquely expressed in petals, often at significantly higher levels [4, 23, 24]. In *D. ajacis*, the *DeajAP3–3* is expressed in all the petal primordia before their morphological differentiation at earlier stages, but is exclusively expressed in the dorsal spurred petals at later stages [4, 28]. Our study in *D. anthriscifolium* largely corroborates these findings, revealing a preferential and persistent expression of *AP3–3* in the petals, and most notably in the dorsal spurred and lateral flat petals throughout the developmental stages (Figs 2B and 3).

Despite the conservativeness of the *AP3–3* expression in *Delphinium*, their functions showed not only the similar but also distinct roles. For instance, in *D. ajacis*, knockdown of *DeajAP3–3* resulted in the transformation of dorsal petals into double-spurred sepals [4]. Similarly, in our study, the pivotal role of *AP3–3* in petal identity is further supported by VIGS experiments, where silencing of *DeanAP3–3* induced a complete morphological reversion of petals to non-spurred sepals (Fig. 4B; Fig. S3, see online supplementary material). These results suggest that the *AP3–3* orthologs have conserved roles in the determination of the dorsal petal identity. Despite the functional conservation of *AP3–3*, it exhibits some non-conservative feature across different species. For instance, in *D. ajacis*, knockdown of *DeajAP3–3* resulted in the transformation of the fused single-spurred dorsal petal into a fused double-spurred sepal [4], while in *DeanAP3*–*3* silenced *D. anthriscifolium* flowers, the spurred petals transformed into non-spurred sepals, suggesting a potential association with *CYC*-like genes that determine floral symmetry. In addition, the *D. ajacis DeajAP3–3* gene specifically determines the identity of spurred petals, as only the dorsal spurred petals transformed into sepals upon its silencing, and the reduced petals remain unchanged. While in *D. anthriscifolium*, the *DeanAP3–3* gene determines the identity of all types of petals, as spurred, flat, and reduced petals all transformed into sepals.

In plants, alterations in gene expression patterns in time, space and amount play a pivotal role in the morphological diversification of both vegetative organs (such as roots, stems, and leaves) and reproductive organs (such as flowers in angiosperms). For instance, the expression of Class 1 *KNOTTED1-LIKE HOMEOBOX* (*KNOX1*) genes after leaf primordia initiation determines the formation of simple or compound leaves [29, 30]. Similarly, in various species within the Liliaceae, Orchidaceae, and Ranunculaceae families, the expansion of expression domains of *AP3* and *PI* genes can lead to the development of petaloid structures in the outer whorls of floral organs [13, 31, 32]. Among the various changes in gene expression patterns, changes in expression levels may be the most significant, but least understood [33]. In *Arabidopsis* and its close relatives, differences in the expression levels of the *SHOOT MERISTEMLESS* (*STM*) gene explain variations in leaf serration or absence among different species [34]. In the Ranunculaceae, the presence or absence of petals is closely associated to the expression levels of the *AP3–3* gene, which exhibits specific and high expression in species with petals, while in species without petals, it is either not expressed or expressed at low levels [16, 24, 26].

Interestingly, as a petal identity gene, the changes in the *AP3–3* gene expression levels also significantly influence the morphological differentiation of petals. For instance, in Nigelleae species, a close relative of Delphinieae, the *AP3–3* gene is consistently expressed at a similar level in all eight non-spurred petals, indicating its role in maintaining a consistent petal phenotype [11]. Complete *AP3–3* silencing results in all the petals transforming into sepals, while partial silencing produces a range of petal phenotypes, from an intermediate to sepal-like or petal-like forms, suggesting a connection between morphological changes in *Nigella* petals and *AP3–3* expression levels [11]. Within Delphinieae, *AP3–3* expression gradients are thought to contribute to the petal differentiation along the dorsoventral floral axis, with species like *Delphinium exaltatum* and *Staphisagria picta* exhibiting higher *AP3–3* expression in dorsal spurred petals [4, 24, 28]. In this study, we also confirmed that the expression level of the *DeanAP3–3* gene is higher in dorsal spurred petals compared to lateral flat petals. From the S10–S12, a critical phase for morphological differentiation between dorsal and lateral petals, the *DeanAP3–3* gene consistently maintained higher expression levels in spurred petals than flat petals. No expression signal was detected in degenerated petals during this period (Fig. 3E–F). In addition, when the *DeanAP3–3* gene is not completely silenced, dorsal spurred petals transform into flat petals, or take an intermediate form between sepals and flat petals, or resemble flat petals, while ventral reduced petals transform into sepals (Figs 4C–F and 5A–C; Fig. S3, see online supplementary material). Therefore, we can conclude that the morphological differentiation of *D. anthrisfolium* petals is influenced by changes in *AP3–3* gene expression levels.

## Materials and methods

### Plant materials and growth conditions

Seeds of *D. anthriscifolium* were collected from Taibai Mountain, Shaanxi, China, and sowed in a soil mixture (vermiculite: nutrient soil = 1:1). The plants were cultivated under controlled conditions, with a photoperiod of 14 hours of light at 20000 lux and 10 hours of darkness, at a temperature of 24°C and a relative humidity of 50–60%.

### Morphological and developmental observations

Typical mature flowers were photo-documented using an EOS 760D digital camera (Canon, Tokyo, Japan) paired with a Canon EF-S 60 mm f/2.8 lens. Floral organs were dissected under an Olympus SZX16 stereomicroscope (Olympus, Tokyo, Japan) and imaged with an OLYMPUS SC180 digital camera. Micromorphological observations were performed as previously described [4].

For scanning electron microscopy (SEM) analysis, petals were dissected under a stereomicroscope, followed by dehydration through a graduated series of water-ethanol solutions. Subsequently, they were dried using a CO_2_ critical-point dryer. Once prepared, the samples were mounted on aluminum stubs, subjected to gold sputter coating, and observed under a S-3400 N scanning electron microscope (Hitachi, Tokyo, Japan).

### Gene isolation and phylogenetic analysis

Total RNA was extracted from floral buds using the SV Total RNA Isolation System (Promega) and reverse-transcribed into cDNA using the thermo scientific RevertAid First Strand CDNA Synthesis Kit. The CDS sequence of *DeanAP3*, *DeanAGL6*, and *DeanPI* lineage genes was then amplified with specific primers (Table S1, see online supplementary material). The PCR products of the expected lengths were inserted into the pEASY®-Blunt E1 cloning vector (TransGen) and confirmed by sequencing.

Candidate *AP3*-like, *AGL6*-like, and *PI*-like genes of other species were obtained from the publicly available databases through BLAST searches (File S1, see online supplementary material). Nucleotide sequences for each gene lineage were aligned and manually adjusted using MEGA X [35]. The alignable nucleotide matrix was utilized for phylogenetic reconstruction in MEGA X, employing the maximum-likelihood method with the best-fit model of JTT-G. Analysis was carried out using 1000 bootstrap replicates. Trees were rooted with genes from early diverging angiosperms.

### Gene expression studies

The reference transcriptome and digital gene expression (DGE) profiles were obtained from published data (GSE249849) [36]. The DGE profiles included 28 samples with three biological replicates each. These samples encompassed seven organ types (dorsal spurred petals, lateral non-spurred petals, dorsal spurred sepals, lateral sepals, ventral non-spurred petals, stamens, carpels), each collected at four developmental stages (S9, S10, S12, S16). Three biological replicates of each were collected. The clean reads of DGE profiles were separately mapped to the reference transcriptome we previously assembled by TopHat2 and fragments per kilobase per million mapped reads (FPKM) values were calculated using RSEM [37, 38].

The expression patterns of *DeanAP3–3* were revealed through mRNA *in situ* hybridization. Floral buds at various developmental stages of *D. anthriscifolium* were fixed in fresh 4% paraformaldehyde and embedded in Paraplast Plus (Sigma) [11]. The antisense probe for *DeanAP3–3* was synthesized via RNA transcription using the *DeanAP3–3*-R primer containing the T7 promoter in conjunction with the *DeanAP3–3*-F primer (Table S1, see online supplementary material).

### Gene functional studies

VIGS was used to study *DeanAP3–3*’s function following Wang *et al.* (2016) [11]. Gene fragments were added to the TRV2-based pYL156 vector and introduced into *Agrobacterium tumefaciens* GV3101. VIGS vectors for *DeanPDS* and *DeanAP3–3* were created using primers for *DeanPDS* and *DeanAP3–3* (Fig. S2B; Table S1, see online supplementary material). TRV2-*DeanPDS*-*DeanAP3–3* was used for at least three treatments, with *TRV2-DeanPDS* as a mock. Flower changes were observed, and silencing efficiency was assessed by qRT-PCR. Total RNA extraction from petals and floral buds of wild-type and VIGS-treated plants at S12 was performed using Trizol® reagent following the protocol (Invitrogen). The integrity and quality of total RNA were evaluated using a 1% agarose gel, ND-2000 (NanoDrop Technologies, Wilmington, USA) and Agilent 2100 Bioanalyzer (Agilent Technologies, Santa Clara, USA) (Table S2, see online supplementary material). Primers *Actin*-qRT and *DeanAP3–3*-qRT were used to assess *DeanAP3–3* expression in VIGS-treated plants (Table S1, see online supplementary material) using a quantitative RT-PCR (qRT-PCR) experiment.

## Acknowledgements

We would like to thank Dr. Hongzhi Kong and Hongyan Shan for valuable comments and suggestions, and the editors and the two anonymous reviewers for their insightful suggestions. Some texts in this paper were polished by Stork’s Writing Assistant (https://www.storkapp.me/writeassistant/). This work was supported by National Natural Science Foundation of China grant (32372750 and 31970247), Qin Chuangyuan High-level Innovation and Entrepreneur-ship Talent Program (2021QCYRC4-51), and the Fundamental Research Funds for Northwest A&F University.

## Author contributions

R.Z., L.G., and P.Z. conceived and designed the experiments. P.Z., Y.X., W.X., L.L., H.Z., and R.Z. collected the plant samples. P.Z., H.Z., Y.X., L.L., and W.X. performed the experiment. P.Z. and L.G. analysed the data and performed the visualization. L.G., R.Z., and P.Z. contributed to the writing and revision of the manuscript. All authors read and approved the final paper.

## Data availability

All data supporting the conclusions of this study may be found in the publication and its supplemental materials, which are available online. Any additional relevant information can be obtained from the corresponding authors upon request (Liping Guo and Rui Zhang).

## Conflict of interest statement

The authors declare no conflict of interest.

## Supplementary data

Supplementary data is available at *Horticulture Research* online.

## Supplementary Material

Web_Material_uhae097
