# Supplementary material for: Roles of the APETALA3–3 ortholog in the petal identity specification and morphological differentiation in Delphinium anthriscifolium flowers
Source: Hortic Res. 2024 Apr 9;11(6):uhae097. doi: 10.1093/hr/uhae097 (PMC11161261; doi:10.1093/hr/uhae097)
Supplement: Web_Material_uhae097 [file web_material_uhae097.zip › Supplemental Figure S1.pdf]

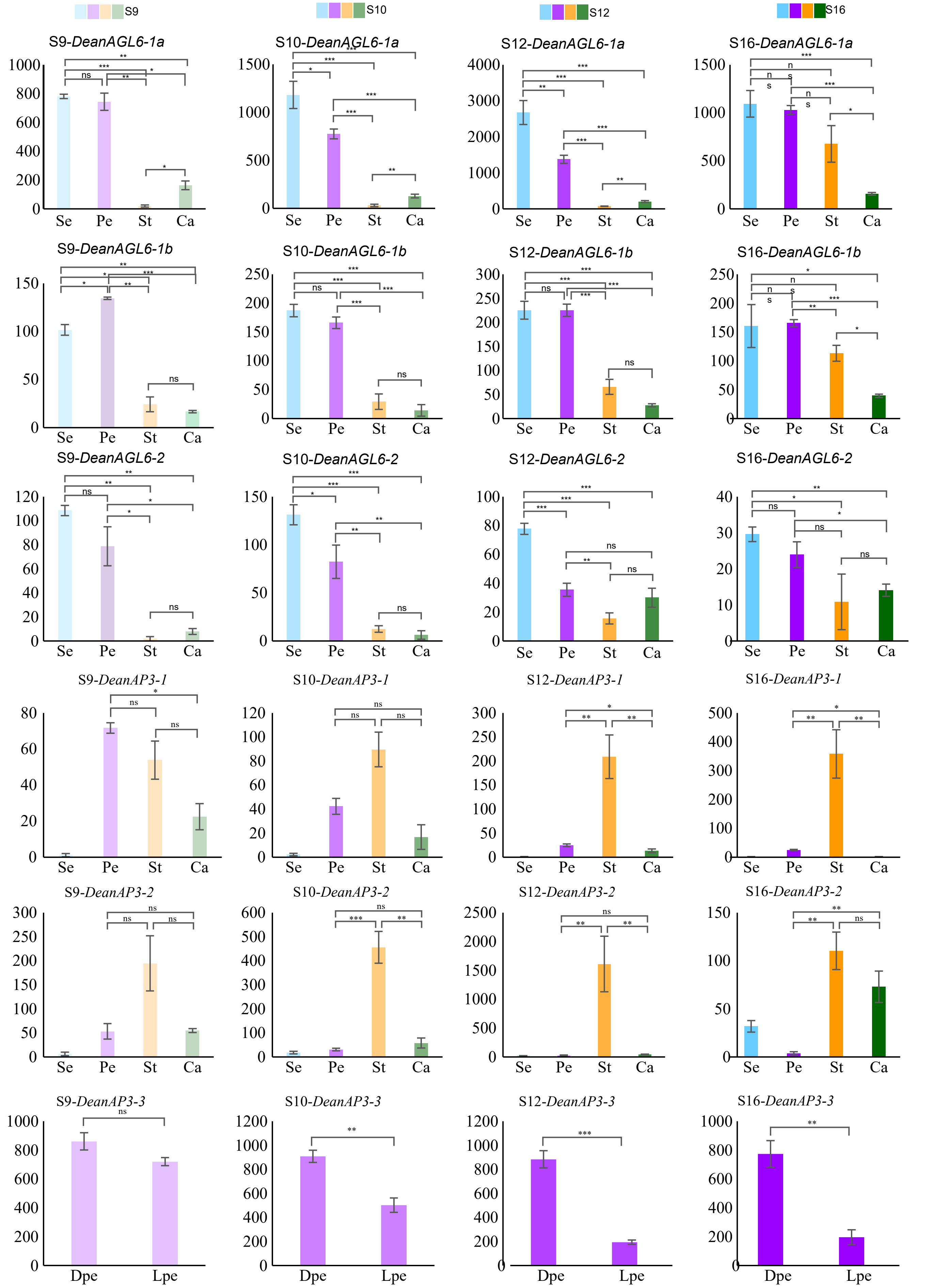


**Figure S1. Spatiotemporal expression patterns of *AGL6*, *AP3*, and *PI* lineage genes as determined by DGE analysis.** The top inset each column depicts sepals (blue), petals (purple), stamens (orange), and carpels (green) across developmental stages S9-S16. ns, $p > 0.05$; *, $p < 0.05$; **, $p < 0.01$; and ***, $p < 0.001$.
