# Supplementary material for: Roles of the APETALA3–3 ortholog in the petal identity specification and morphological differentiation in Delphinium anthriscifolium flowers
Source: Hortic Res. 2024 Apr 9;11(6):uhae097. doi: 10.1093/hr/uhae097 (PMC11161261; doi:10.1093/hr/uhae097)
Supplement: Web_Material_uhae097 [file web_material_uhae097.zip › Supplemental Figure S2.pdf]

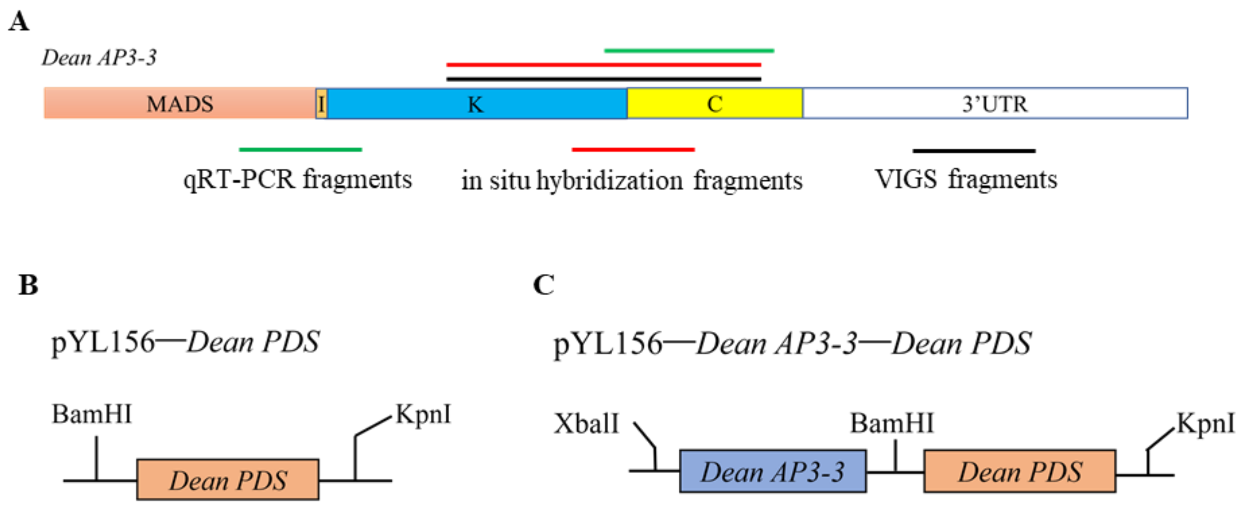


**Figure S2. VIGS constructs of *DeanAP3-3* gene**. (A) Sequence features of *DeanAP3-3* gene. Regions encoding the MADS, I, K and C domains are highlighted by boxes with different colors. The fragments for the qRT-PCR (green), *in situ* hybridization (red), and construction of VIGS vectors (black) are indicated by the corresponding colored lines. (B-C) Schematics showing the arrangement of VIGS fragments in different constructs.
