# Supplementary material for: Roles of the APETALA3–3 ortholog in the petal identity specification and morphological differentiation in Delphinium anthriscifolium flowers
Source: Hortic Res. 2024 Apr 9;11(6):uhae097. doi: 10.1093/hr/uhae097 (PMC11161261; doi:10.1093/hr/uhae097)
Supplement: Web_Material_uhae097 [file web_material_uhae097.zip › Supplemental Figure S3.pdf]

A

Constuct	Flower	Number of organs: Dorsal sepal	Lateral- ventral sepal	Dorsal petal	Lateral petals	Ventral petal	Stamen	Carpel	All	Type
TRV2–*DeanPDS*–*DeanAP3-3*	I-1-1	1	4	2	2	3	8	3	23	Strong
	I-1-2	1	4	2	2	4	6	3	22	
	I-2-1	1	4	2	2	3	11	3	26	
	II-1-1	1	4	2	2	4	8	3	24	
	II-2-1	1	4	2	2	4	8	3	24	
	II-2-2	1	4	2	2	4	8	4	25	
	III-1-1	1	4	2	2	4	9	3	25	
	7 more flowers showing similar phenotypic changes									
	I-3-2	1	4	2	2	2	10	4	25	Moderate
	I-4-2	1	4	2	2	0	10	3	22	
	II-4-1	1	4	2	2	0	8	3	20	
	III-1-2	1	4	2	2	2	9	3	23	
	5 more flowers showing similar phenotypic changes									
	I-1-3	1	4	2	2	1	7	3	20	Weak
	II-1-4	1	4	2	2	0	9	3	21	
	III-1-1	1	3	2	2	0	8	3	21	
	III-2-1	1	4	2	2	0	7	3	19	
	…………									

B

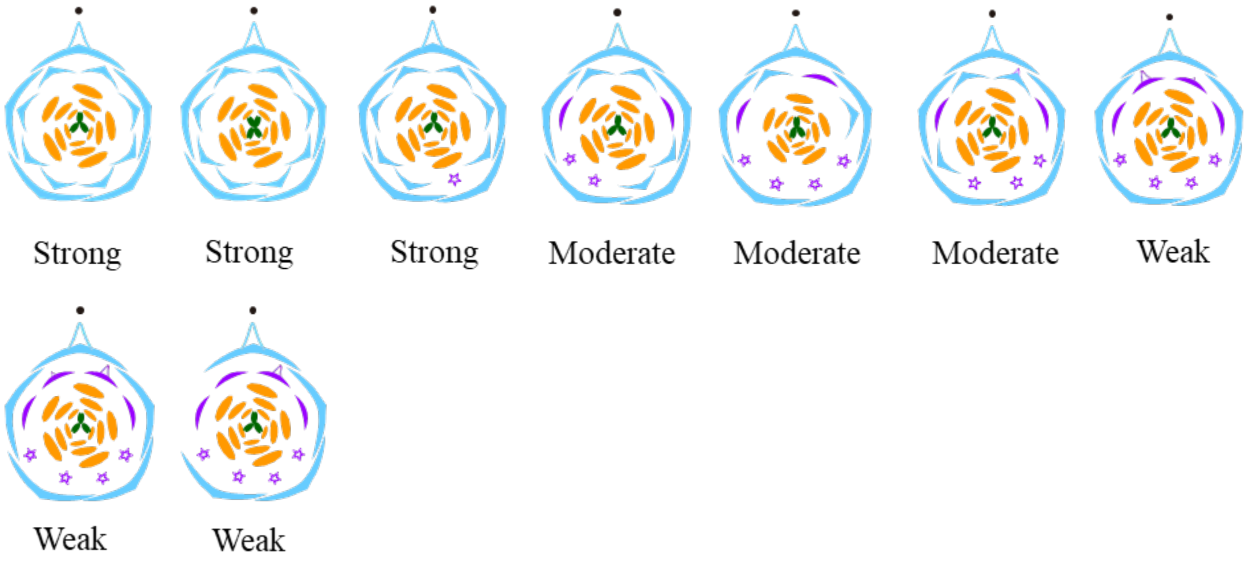

Figure S3. Flowers of TRV2-*DeanPDS-DeanAP3-3* treated plants. (A) Number of floral organs in VIGS-treated flowers (batch #_ inflorescence #_flower #). (B) Floral diagrams of representative types.
